# Comparison of Microbiological and High-Performance Liquid Chromatographic Methods for Determination of Clarithromycin Levels in Plasma

**Published:** 2010

**Authors:** Farzaneh Lotfipour, Hadi Valizadeh, Somayeh Hallaj-Nezhadi, Morteza Milani, Parvin Zakeri-Milani

**Affiliations:** a *Faculty of Pharmacy, Tabriz University of Medical Sciences, Tabriz, Iran.*; b * Drug Applied Research Center, Tabriz University of Medical Sciences,Tabriz, Iran. *

**Keywords:** Clarithromycin, Microbiological assay, High-performance liquid chromatographyPlasma, Bioassay method

## Abstract

An agar well diffusion bioassay method for determination of clarithromycin in human plasma, using *Micrococcus Luteus *ATCC 9341 as the assay organism, was compared with a selective high-performance liquid chromatographic (HPLC) method with UV detection. Spiked plasma was used to prepare standard and control samples for both methods. The results of the bioassay analyses with spiked plasma samples were concordant by HPLC methods (R^2^ =0.871, P < 0.001).The Bland-Altman method also showed good agreement between the results of two methods. HPLC demonstrated an improved precision (0.88-19.86% versus 4.51-26.78%) and accuracy (99.27-103.42 % versus 78.52-131.19 %), compared to those of the bioassay method. The range of linearity obtained by both methods (from 62.5 to 3000 ng/ml for HPLC and from 250 to 3000 ng/ml for the bioassay) includes the range of concentrations of clarithromycin which are considered clinically relevant. However, comparison between HPLC and microbiological assays after oral administration of clarithromycin in healthy volunteers indicated significant differences between the two methods in mean plasma concentration–time profiles. The Bland-Altman method revealed no agreement between the two methods, which can be explained by the presence of active metabolites of clarithromycin in plasma.

## Introduction

**Figure 1:**
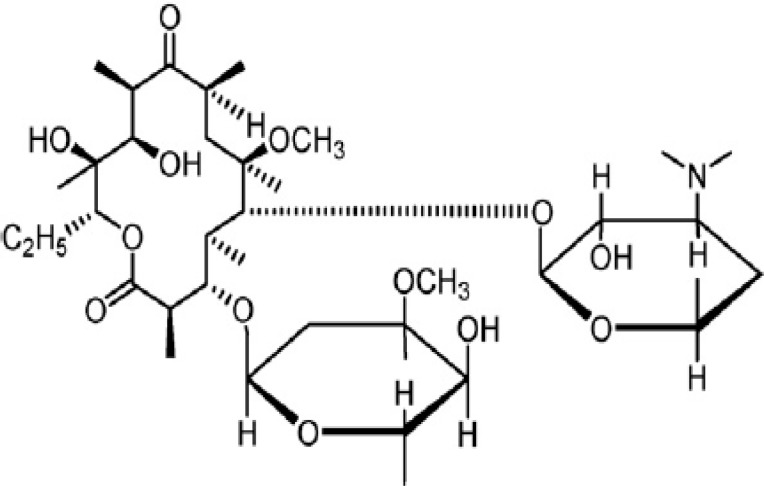
Molecular structure of clarithromycin (2).

Clarithromycin (Figure 1) is a semisynthetic macrolide antibiotic derived from erythromycin A and consists of a 14-membered macrocyclic lactone ring with sugars linked via glycosidic bonds. All macrolides display similar antibacterial properties and constitute an important alternative for patients exhibiting penicillin sensitivity and allergy (1, 2). Clarithromycin is reported to be more active than erythromycin against susceptible streptococci and staphylococci and some other species. Clarithromycin is used in infectious conditions like respiratory-tract, skin and soft-tissue infections as well as leprosy and opportunistic mycobacterial infections (3). Several methods have been reported to detect and quantify clarithromycin concentration in biological fluids, with the majority of them having used high-performance liquid chromatography (HPLC) with detection oriented derivatization (4-6), electrochemical (7-10), amperometric(11) and mass spectrometric detection (12-14). The microbiological assay can be considered as an alternative method to HPLC. This assay can reveal subtle changes not demonstrable by conventional chemical methods. Moreover, microbiological assay requires not only no specialized equipment but also no toxic solvents (15). Concordance between the bioassay and specific HPLC methods for clarithromycin has not been investigated in a precise manner. Hence, this study was set out to provide such data using spiked plasma as well as plasma samples collected from healthy volunteers after oral dosing of clarithromycin.

## Experimental

*Preparation of samples*

Plasma samples used were from six healthy male volunteers aged between 21 and 37 years and weighing from 56 to 90 Kg. None of them had a history of hypersensitivity to medications. This study was carried out in accordance with the guidelines of the Declaration of Helsinki (World Medical Assembly 1964) as revised in Edinburgh. The study protocol was approved by the ethics committee of Tabriz University of Medical Sciences. An equivalent 500-mg dose of clarithromycin (Klacid^®^ suspension, containing 125 mg of clarithromycin per 5 mL, Abbott Laboratories LTD, Italy. Batch number: 38398TF01) was given orally to each subject as a single dose with 200 mL of water. The subjects were allowed to have breakfast (a snack) after two hours. Five milliliters of blood were drawnat 0, 0.5, 1, 1.5, 2, 3, 4, 6, 8, 10, 12 and 24 h after each administration. The blood samples were taken from subject’s forearm veins. All samples were centrifuged in heparinated tube. The plasma samples were separated and kept frozen at a temperature below -20 °C for subsequent analysis.

*I) Bioassay*

*Preparation of references substance*

A quantity of clarithromicin reference substance (Elder Pharmaceuticals LTD, India) equivalent to 50 mg of clarithromicin was accurately weighed and transferred to a 50 mL volumetric flask. Methanol was added to make up volume in order to give a final concentration of 1 mg/mL. From this solution, the stock concentration of 3000 ng/mL in blank plasma was prepared and aliquots of this stock were diluted with the blank plasma to obtain various concentrations of 3000, 2000, 1000, 500 and 250 ng/mL, which were used in the assay. 

*II) Microorganism and inoculums standardization*

*Micrococcus luteus *ATCC 9341 was purchased in lyophilized form (Pasteur Institute, Iran) and activated in Tripticase Soy broth medium. Fifty microliters of the growth medium was transferred into antibiotic agar medium I (24 h before assay) and incubated at 35 °C for one day. The bacterial growth culture was diluted with a 0.9% w/v saline solution, in order to reach 30% turbidity at 580 nm, and the resultant bacterial suspension was then used as culturing inoculums. 

*III) Well diffusion assay*

Microbiological assay of clarithromycin was performed, using an agar well diffusion procedure. Briefly, the assay plate contained 25 mL of antibiotic agar I inoculated with the bacterial inoculums. Wells of 6mm diameter were punched and filled with 100 μl of calibration samples or test samples. After 24 h of incubation at 35°C, the diameter of the inhibition zone was measured. The method was validated by determination of the following operational characteristics: linearity, precision and accuracy. The linearity was evaluated using the linear regression analysis, which was calculated by the least squares regression method. One-hundred microliter samples of clarithromycin reference solution were added as concentrations of 3000, 2000, 1000, 500 and 250 ng/mL into the spiked plasma samples. Each level was made in triplicate and employed on the well diffusion assay method described above. The precision of the assay method was determined by evaluating repeatability (intra-assay) and intermediate precision (inter-assay), and expressed as the relative standard deviation (RSD) of four quality control samples. The accuracy was determined by adding known amounts of clarithromycin reference substance (quality control samples) at the beginning of the process, followed by calculation of the value: measured value/nominal value × 100. 

**Figure 2:**
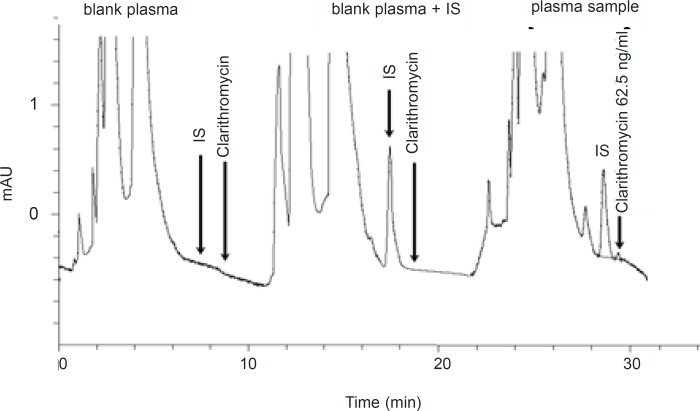
Representative chromatogram of a typical blank plasma sample and different clarithromycin standard concentrations in plasma samples

*HPLC method*

The analytical procedure for determination of clarithromycin in plasma was adopted from the method presented by Amini and Ahmadiani (6). The method used was validated for specificity, accuracy, precision and sensitivity. Fifty microliters of the internal standard (1 μg/m1 of diltiazem HCl) and 20 microliters of 1 N NaOH were added to 1 mL of plasma. The mixtures were extracted with 2.5 mL hexane:isopropyl alcohol (98:2 %v/v), by vortexing for 5 min. After centrifugation for 5 min at 1000 g, the upper organic phase was transferred to a 5 mL glass tube and 50 microliters of 0.2% acetic acid was added. The mixture was vortex-mixed for 2 min and then part of the upper organic phase was discarded and the remaining mixture (about 1 mL) transferred into a 1.5 mL microcentrifuge tube. After centrifugation for 2 min, the upper organic phase was discarded completely. Finally, 50 microliters of the aqueous phase was injected onto the HPLC column. The mobile phase consisted of acetonitrile and 50 mM aqueous sodium dihydrogen phosphate (32:68 %v/v), with pH=4.5 (adjusted with concentrated phosphoric acid and 4M sodium hydroxide).The analytical column used for chromatographic separations was Shimpack CLC-CN 5 μm (250 × 4.6 mm), with a Shimpack CLC-CN 5 μm 4.6 × 20 mm guard column. The flow rate was 1 mL/min at 40°C and the detector wavelength was set at 205 nm. Under these conditions, the retention times for clarithromycin and the internal standard (diltiazem) were 8.6 and 7.5 min, respectively. The liquid chromatographic system (Knauer, Germany) consisted of a Knauer K1000 solvent delivery module equipped with a Rheodyne (Cotati, CA) injector and a variable wavelength ultraviolet spectrophotometric detector (Knauer smartline 2500). Eurochrom 2000 version 2.05 was used for data acquisition, data reporting and analysis. All plasma samples taken from each volunteer during the two treatment periods, were analyzed in the same chromatographic run (analytical own control). Each run had a separate daily calibration. Quality control samples (QC) at two different concentration levels were used in each run. Calibration curves were obtained by plotting the clarithromycin to diltiazem peak area ratio, against the concentrations of the standard solutions.

*Statistical methods*

The least-squares linear regression was performed, using the standard techniques. Both procedures were repeated over 4 days, and each control was run four times. The within- and between-run precisions of the assays were estimated by computing the Relative Standard Deviation (RSD). The agreement between both analytical methods was evaluated by computing the mean square error and the Bland-Altman method (16). 

## Results

*Validation of the microbiological method*

**Figure 3:**
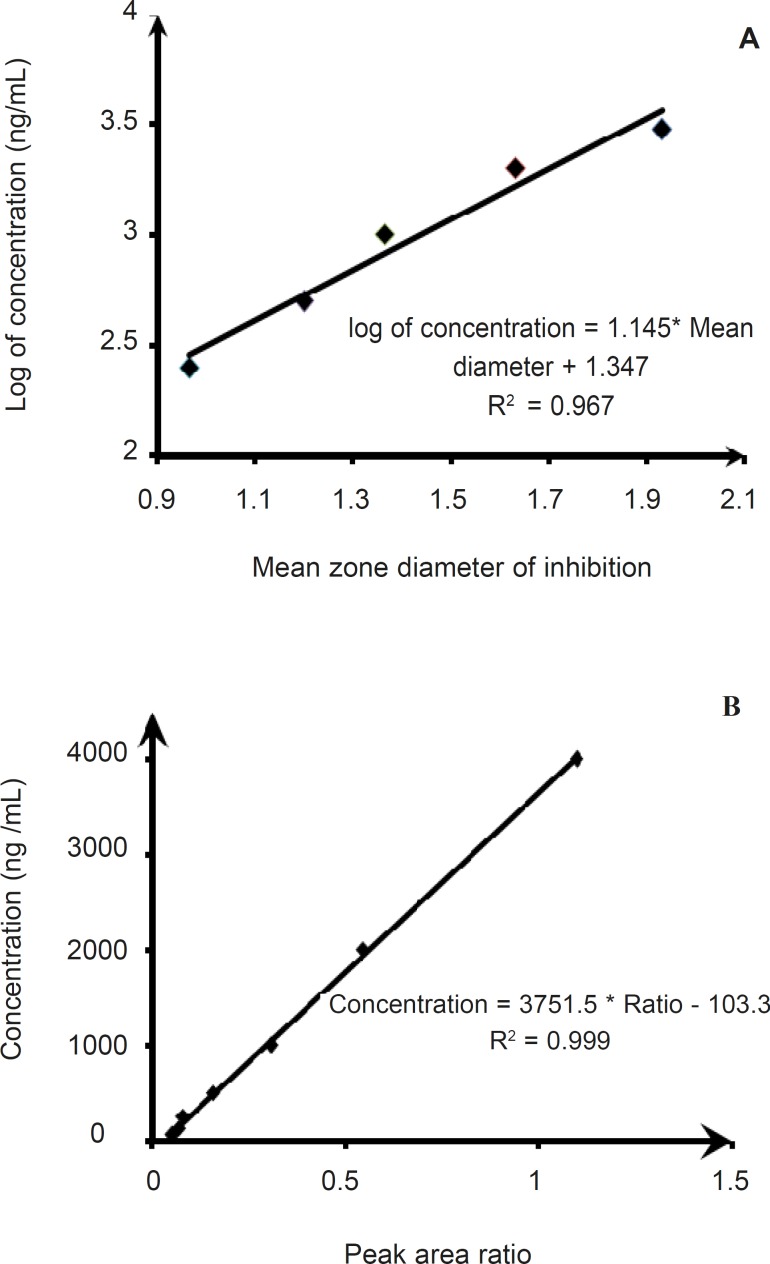
Calibration curves for clarithromycin obtained by the microbiological (A) and HPLC (B) assays.

Based on USP, the reference microorganism for assaying erythromycin is *Micrococcus Luteus *(17). This bacterium is non-pathogenic and showed a rapid growth at 35°C, and also has good sensitivity to clarithromycin. Hence, it was selected in this study. The regression analysis of clarithromycin data generated a linear curve with a correlation coefficient of 0.967 (Figure 3A). Intra-assay precision was determined to be 12.86 - 24.49 % (Table 1). Inter-assay variability was calculated from assays on 3 days and shows RSD volues. of 4.51 to 26.78% (Table 1). The accuracy was found to be 82.57 - 138.02% and 78.52 to131.19 % for intra-assay and inter-assay, respectively (Table 1). 

*Validation of the HPLC method*

Calibration curves were obtained by plotting the clarithromycin to diltiazem peak area ratio, 

against the concentrations of the standard solutions. The method was linear within the 62.5 to 3000 ng/mL range, with a coefficient of correlation (R^2^) greater than 0.999 (Figure 3B). The LOQ was 62.5 ng/mL of clarithromycin plasma concentration repeatability, with a within-day repeatability of ± 0.8 % for a concentration of 500 ng/mL and a day-to-day repeatability of ±2.0% for the same concentration. The accuracy was found to be 90.41-99.74%, and 99.27-103.42% for intra-assay and inter-assay, respectively (Table 1).

**Table 1:** Nominal and determined concentrations of quality control (QC) samples by microbiological and HPLC assay methods

**Assays **		**Nominal concentration (ng/ml)**				
		250	500	1000	2000	3000
**Microbiological Intra-assay **	Mean concentration obtained (ng/ml) (*n*=3)	345.06	600.59	873.98	1651.43	3261.38
	Precision (SD)	44.36	138.60	112.37	404.46	452.97
	Precision (RSD, %)	12.86	23.08	12.86	24.49	13.89
	Accuracy	138.02	120.11	87.39	82.57	108.71
**Microbiological Inter-assay **	Mean concentration obtained (ng/ml) (*n*=3)	327.99	464.32	870.53	1570.46	2944.52
	Precision (SD)	14.79	124.32	151.93	395.31	786.25
	Precision (RSD, %)	4.51	26.78	17.45	25.17	26.70
	Accuracy	131.19	92.86	87.05	78.52	98.15
**HPLC Intra-assay **	Mean concentration obtained (ng/ml) (*n*=3)	248.57	492.14	990.74	1994.82	2712.25
	Precision (SD)	10.59	4.33	17.08	10.95	538.64
	Precision (RSD, %)	4.26	0.88	1.72	0.55	19.86
	Accuracy	99.43	98.43	99.07	99.74	90.41
**HPLC Inter-assay **	Mean concentration obtained (ng/ml) (*n*=3)	258.56	496.34	1002.85	2002.43	3011.78
	Precision (SD)	13.15	9.03	16.17	15.11	7.80
	Precision (RSD, %)	5.08	1.82	1.61	0.75	0.26
	Accuracy	103.42	99.27	100.28	100.12	100.39

*Comparison of the methods*

*I) Correlation of HPLC and Bioassay Results in Spiked Plasma Samples*

**Figure 4:**
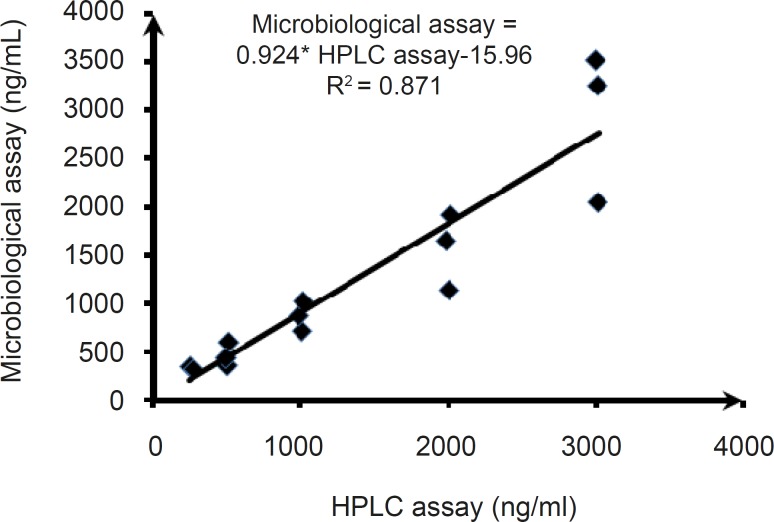
Correlation between concentration obtained by microbiological and HPLC assays

**Figure 5:**
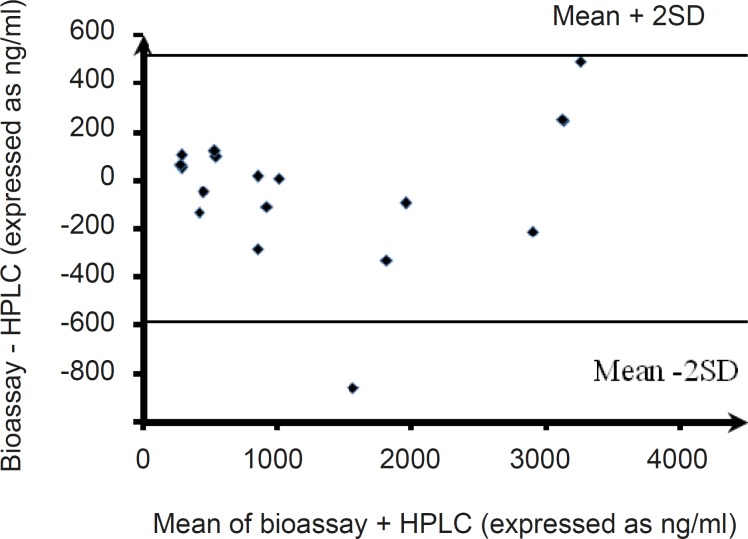
Bland-Altman plots of the agreement of plasma level measurements by the bioassay and HPLC methods (the difference between the bioassay and HPLC data sets vs. the mean of the two methods for pooled plasma clarithromycin samples are shown).

The scatter plot of concentrations of clarithromycin in spiked plasma samples obtained by the microbiological assay versus those observed from the HPLC assay (Figure 4) demonstrated a dose-dependent relationship, with a regression coefficient of 0.871 (P < 0.001) and a slope of 0.924. The slope of scatter plot indicated that values estimated using the HPLC method were about 8% higher than those obtained from the microbiological assay method. Finally, the Bland-Altman plot was used to measure the agreement of the results obtained from the clarithromycin bioassay and the HPLC method. The Bland-Altman plot illustrates the differences between the bioassay and HPLC data sets versus the mean of clarithromycin concentrations obtained using these two methods. The mean difference in concentrations gained by the two methods was -33.94, with limits of agreement of -588.44 and 512.64 (Figure 5). This indicates an agreement between the HPLC and bioassay methods for determining the clarithromycin level in spiked plasma samples, with 95% of the differences lying between the limits. 

*II) Correlation of hplc and bioassay results following clarithromycin administration*

**Figure 6:**
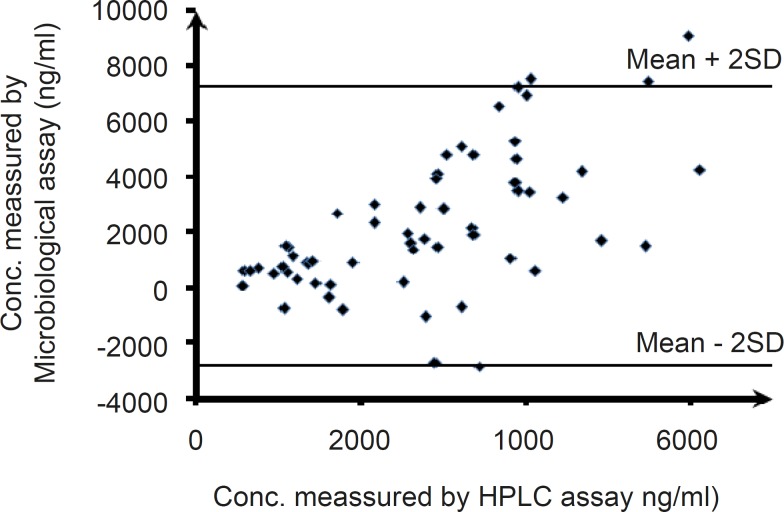
Bland-Altman plots of the agreement of plasma level measurements using the bioassay and HPLC methods (the difference between the bioassay and HPLC data sets vs. the mean of the two methods following clarithromycin administration are shown).

Correlation between the mean concentration of the samples after oral administration of clarithromycin, which related to various time intervals determined by the microbiological and HPLC methods, indicates a significant difference between the two methods with an unacceptable regression coefficient (data not shown). The differences between the bioassay and HPLC data sets versus the mean of clarithromycin concentrations obtained after oral administration of the drug using the two methods, were also plotted. Based on the results obtained the mean of the differences was 2225.6, with limits of agreement lying between -2809.2 and 7260.4 (Figure 6). Having less than 95 % of the differences lying between these limits of -2809.2 and 7260.4, it can be concluded that the two methods do not agree with each other, based on the Bland-Altman plot. 

**Figure 7:**
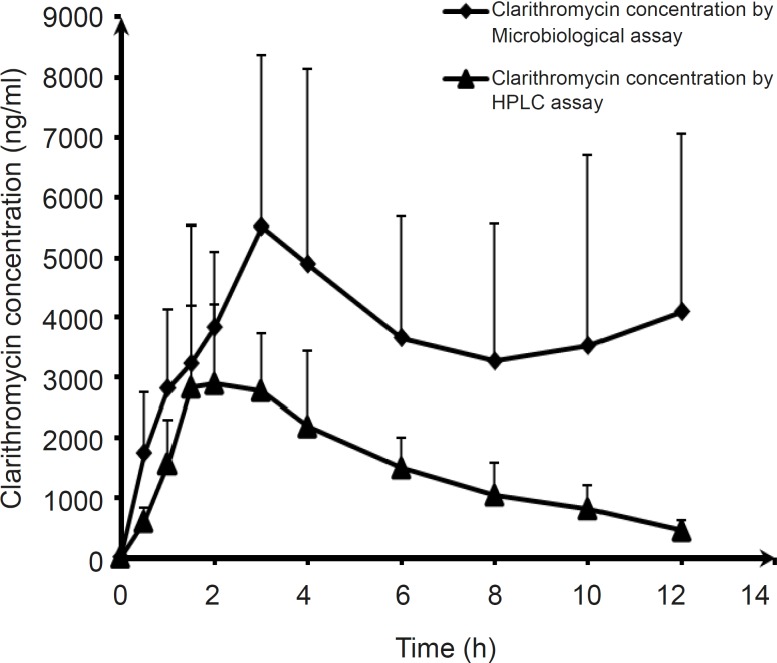
Mean plasma concentration–time profiles of clarithromycin (Klacid) by microbiological and HPLC assay in healthy volunteers, following a 500-mg dose of clarithromycin suspension

The mean plasma concentration–time profiles following oral adminstration of clarithromycin, measured by microbiological and HPLC assays, have been shown in Figure 7. As it can be seen, the plasma concentrations of the drug at various time intervals determined using microbiological method were significantly higher than those measured by the HPLC method. Also, AUC (0-12 h) values of clarithromycin for the individual subjects, obtained by microbiological and HPLC assays, were compared and shown in Table 2. It could be seen that the figures obtained by microbiological assay are markedly higher. These differences have a noticeably increasing trend, as reaching the end point of the graph. Predictably, the mean area under the plasma concentration–time curve calculated using the HPLC and the microbiological assay methods also indicated remarkable differences (discrepancy as high as 3.2 times overestimation by the microbiological assay method). This could be ascribed to the presence of active metabolite of clarithromycin. 

**Table 2:** Comparison of AUC (0 –12 h) values of clarithromycin (Klacid^®^) for the individual subjects by microbiological and HPLC assays.

**AUC and ratio **	**Subject**						**Mean**	**RSD (%)**
	**A**	**B**	**C**	**D**	**E**	**F**		
**AUC (microbiological) **	68954.26	70237.49	47135.81	18103.15	18717.29	63607.54	47792.59	50.64
**AUC (HPLC) **	18311.58	11246.59	10910.35	23158.33	24657.48	19091.18	17895.92	32.39
**AUC ratio (microbiological: HPLC)×100 **	376.56	624.52	432.03	78.17	75.91	333.18	3.20	66.53

## Discussion

When comprising the bioassay and HPLC methods, each exhibits several advantages and deficiencies. For bioassays, important advantages are inexpensiveness, simplicity and fastness (18, 19), whereas low specificity of the bioassay methods can be considered as their main disadvantages. Since, bioassay determines all antimicrobial compounds including metabolites that show antimicrobial activity in serum or plasma, it could be used to investigate the biological properties of metabolites. Therefore, bioassay should be used in parallel and not to replace highly specific methods, such as HPLC (20). On the other hand, the non-specific nature of bioassay is a great disadvantage, especially when the investigation of pharmacokinetic of only the parent compound is considered. Among the other problems of the microbiological assay method is its lower sensitivity than the instrumental methods such as the HPLC method. Advantages of the HPLC method are its excellent precision and specificity (18). 

Since microbiological method can show the pharmacological effect of some compounds like antibiotics, vitamins and amino acids, it is usually used to determine these groups of compound in biological fluids. Comparison of microbiological and the instrumental methods (like HPLC) for determination of drug concentrations in plasma or other biological methods are routinely performed to investigate the variations in the pharmacological activity of drugs in the biological fluids or the potential pharmacological activity of the metabolites. For example, Kotecka and coworkers developed a bioassay method for artesunate and compared it to the HPLC method. They showed that bioassay of the active drug in plasma correlates well with the specific chemical analysis by HPLC (21). In other studies, HPLC and microbiological assay were compared for determination of voriconazole and posaconazole levels in plasma and the same results were obtained (19-22). On the other hand, in some other studies discrepancies between the two methods have been shown. For example, in the case of itracinazole the difference between the results of the two methods has been shown and explained by metabolism of itraconazole to hydroxyitraconazole, an active metabolite (23). 

For determination of clarithromycin concentration in human plasma, several methods have been established so far, more dominantly HPLC based procedures. Discrepancies between the HPLC method and possible bioassay methods could be predictable, as active metabolite of clariyhromycin has been shown to be more active than the parent compound (3, 24). However, concordance between the two methods for clarithromycin has not been investigated in a precise manner. 

This study compares an HPLC and a bioassay method for the determination of clarithromycin levels in human plasma. The HPLC method with UV detection involves a protein precipitation step with NaOH, hexane/isopropyl alcohol and acetonitrile, followed by HPLC on a CLC-CN column. On the other hand, the bioassay method consists of a simple agar well-based microbiological method that determines clarithromycin concentrations by measuring growth inhibition zones of *M. luteus *and comparing unknown concentrations of clarithromycin in plasma against a standard curve.

Firstly, the results of in vitro analysis of different clarithromycin concentrations in blank plasma (without dosing to the healthy volunteers), using HPLC and bioassay were compared. The results demonstrated a good correlation between the two procedures, as can be seen in figure 4. Also, the agreement between HPLC and bioassay methods for determining clarithromycin in spiked plasma samples (in vitro assay) was shown using the Bland-Altman method, with 95% of the differences lying between the limits of mean ± 2SD ( -588.44 and 512.64). 

On the other hand, when the data pertaining to the concentration of clarithromycin in various time intervals followed by dosing of the antibiotic in healthy volunteers obtained using the two methods were compared, completely different results were observed. As it could be seen in the scatter plot of clarithromycin concentration in various time intervals determined by the microbiological and HPLC assays (Figure 6), less than 95% of the differences were between the limits of mean± 2SD (-2809.2 and 7260.4), indicating a lack of concordance between the two assay methods. This could be explained by the presence of active metabolites of clarithromycin in plasma, as it has been shown that through the metabolism of clarithromycin by CYP3A4, approximately 25% of the systemically bioavailable drug is converted to an active metabolite, 14-OH-clarithromycin (14OHC). has been demonstrated to have significant antibacterial activity against some gram-negative pathogens and in some cases it has been shown to be more active in vitro, as well as in vivo, than clarithromycin (for example against Haemophilus influenza) (25). This could also be confirmed by the mean plasma concentration–time profiles following oral administration of clarithromycin, as measured using both methods. Based on Figure 7, as well as Table 2, the plasma concentration profile of the drug is notably higher (3.2 folds) when measured with the microbiological method, as a consequence of significant antibacterial activity of clarithromycin metabolite.

Based on this finding, it could be concluded that microbiological assay should not be used to replace HPLC method for determining exact level of pure clarithromycin in plasma. However, this method can be used independently to monitor pharmacological activity of clarithromycin in clinical studies, or to complement the HPLC assay method.
